# Neighborhood disorder and depressive symptoms in Jamaican adults: the mediating roles of neighborhood crime and safety problems and collective efficacy

**DOI:** 10.3389/fepid.2025.1467838

**Published:** 2025-04-22

**Authors:** C. Cunningham-Myrie, T. Moore, J. Wiggan, N. Younger-Coleman, S. McFarlane, G. Gordon-Strachan, D. Francis, N. Bennett, I. Govia, M. Tulloch-Reid, T. S. Ferguson, W. Aiken, A. Grant, T. Davidson, K. Webster-Kerr, R. Wilks, K. P. Theall

**Affiliations:** ^1^ Department of Community Health and Psychiatry, University of the West Indies, Mona, Jamaica; ^2^ Department of Social, Behavioral and Population Sciences, School of Public Health and Tropical Medicine, Tulane University, New Orleans, LA, United States; ^3^ National Epidemiology Branch, Ministry of Health & Wellness, Kingston, Jamaica; ^4^ Caribbean Institute for Health Research, University of the West Indies, Mona, Jamaica; ^5^ School of Health and Human Performance, Georgia College and State University, Milledgeville, GA, United States; ^6^ Institute for Global Health, University College London, London, United Kingdom; ^7^ Department of Surgery, Radiology, Anaesthesia and Intensive Care, University of the West Indies, Mona, Jamaica; ^8^ Department of Epidemiology, School of Public Health and Tropical Medicine, Tulane University, New Orleans, LA, United States

**Keywords:** neighborhood disorder, depression, crime and safety problems, collective efficacy, mediation analysis, Jamaica

## Abstract

**Background:**

Neighborhood disorder has been found to be associated with worse mental health outcomes, such as depression. This study examined the association between perceived neighborhood disorder on depressive symptoms in a nationally representative sample of Jamaican adults, and whether any association was mediated by perception of neighbourhood crime and safety problems or collective efficacy (CE).

**Methods:**

Secondary analysis was conducted on the Jamaica Health and Lifestyle Survey (JHLS III). The JHLS III, a cross-sectional nationally representative survey, was administered to 2,807 individuals aged 15 years and older in Jamaica and completed in 2017. Regression analyses were performed to identify associations between perceived neighborhood disorder and depressive symptoms and mediation analyses to examine the roles of perceived neighborhood crime and safety problems and CE in the pathway between perceived neighbourhood disorder and depressive symptomatology.

**Results:**

The odds of depressive symptomatology were 1.55 (95% CI = 1.14, 2.10) times as high among respondents living in neighborhoods perceived as having high disorder compared to those with low disorder. Partial mediation by perceived neighborhood crime and safety problems and low CE in the disorder-depressive symptomatology relation was observed. Twelve percent and 7% of the association between neighbourhood disorder and depressive symptoms were explained through perceived neighborhood crime and safety problems and low CE, respectively. In serial mediation analysis the association between perceived neighborhood disorder and depressive symptoms was mediated by perception of neighborhood crime and safety problems which, in turn, was mediated by reported CE.

**Discussion:**

The pathway between neighborhood disorder and depressive symptoms may be reduced by intervening on reducing neighborhood crime and safety problems and/or improving CE in Jamaican neighborhoods.

## 1 Introduction

Since 1990, depressive disorders have been ranked as the largest contributor to years lived with disability (YLD) in the Caribbean with an additional 948 YLD per 100,000 added in 2013 (1, 2). According to the 2017 Global Burden of Disease database, depressive disorders are among the most common mental health concerns afflicting the population of Jamaica (3). The Jamaica Health and Lifestyle Survey (JHLS III) completed in 2017 (4) revealed that the prevalence of depression was lower when compared to the previous JHLS II (5) completed in 2007/08 (14.3% vs. 20.3%). Nonetheless this remains of concern, given the high prevalence of noncommunicable Diseases (NCDs) in Jamaica and documented higher rates of depression in persons with NCDs (5).

The social, economic, and health condition of individuals are key drivers for depression (6). For instance, significantly higher levels of depression have been reported amongst Jamaicans with Sickle Cell Disease (SCD) compared to controls with normal genotype (7). In a cross-sectional study of adults in rural Haiti, findings revealed employment as a risk factor for depression for both men and women (8). Findings from a cross-national population-based comparative study indicated higher rates of depression in unemployed Jamaicans and Guyanese compared to those who were employed (9). A systematic review assessing studies conducted in Caribbean territories between 2004 and 2014 revealed that the role of social determinants on depression mirrored global findings (6). Specifically, frequency and severity of depression were higher in females, persons with lower occupational levels, income and education, as well as among persons with less social capital (6). In comparison to men, the evidence suggests women are disproportionally affected by depressive disorders (3), with depression being twice the rate for women than men in Jamaica (5). Gender differences were found regarding the association between depression and education, with one Haitian study suggesting higher than a primary school education as a risk factor for depression among women only (8). However, in the aforementioned systematic review (6)**,** education was assessed across 13 articles in which most demonstrated a higher prevalence/depression score among individuals with less education. According to Maharaj et al. (10), in the Caribbean island of Trinidad, individuals with only primary education were almost three times more likely to have depression compared to those with secondary or higher education (OR 2.7, 95%CI 1.4–5.1).

Urbanization is increasing throughout developing countries (11). Despite suggestions that urbanization leads to an enhanced quality of life, urbanization is recognized as a determinant of poor health (12) through various pathways especially for vulnerable groups who are at greater risk of mental health problems (13). Studies examining the intersections of urbanicity and depression have supporting evidence that confirms that people living in urban areas have increased rates of depression (14). In a cross-sectional study conducted in Jamaica, urbanization was found to contribute to the risk of depression symptomatology which differed by sex (15). Specifically, males residing in neighborhoods with poor infrastructure were nearly twice as likely to increase their burden of depressive symptoms (OR 1.92, 95%CI 1.05–3.52) compared to those who lived in neighborhoods with better infrastructure. For women, those who resided in informal communities were twice as likely to increase their burden of depressive symptoms (OR 2.09, 95%CI 1.15–3.82) compared to women who resided in planned communities (15).

Neighborhood characteristics, inclusive of disorder, crime and social cohesion (collective efficacy), have been shown to be associated with health outcomes (16, 17). Neighbourhood disorder refers to social and physical cues indicating lack of order and social control in the community with visible signs of social disorder consisting of contention, drugs, public intoxication, and loitering; physical disorder of neighborhoods consists of vandalism, vacant lots, and cars (17). In the United States, low-and middle-income African Americans perceived neighbourhood social disorder as a risk factor for depression (18, 19). Previous studies conducted in Jamaica have revealed positive correlations between highly disordered neighborhoods and negative health behaviours such as low levels of physical activity and high levels of substance use (20, 21). For example, secondary analysis of the Jamaica National Drug Use Prevalence Survey 2016 revealed that among females, those who perceived higher levels of neighborhood disorder had increased likelihood of alcohol and tobacco use (21). Greater perceptions of neighborhood disorder were associated with higher levels of depressive symptoms among Jamaican adolescents compared with their peers in the Bahamas and St Vincent (22).

Crime has been directly (23) and indirectly (24) associated with depression through perceptions of neighborhood disorder and through experiences of violence in the neighbourhood. Crime rates in Jamaica are among the highest in the world (25) and many neighborhoods are physically and socially disordered (4). According to a review by Mair et al., over 82% (37 out of 45) of observational studies assessing correlations between depression and neighborhood characteristics identified a significant association of one or more neighborhood characteristics with depression or depressive symptoms, also suggesting that the associations were more consistent with social processes such as neighborhood disorder and violence (26).

The relationship between neighborhood disorder and perceived crime and safety problems is complex. Crime and neighborhood disorder both have similar roots that stem from structural characteristics specific to certain neighborhoods such as those with poverty and limited resources (27). Separating neighborhood disorder and crime can be difficult; some aspects of neighborhood disorder are considered crimes. This could include both social and physical disorder, such as soliciting prostitutes and loitering, and incivilities like painting graffiti or other violations of the law (27). Some of these associations are inconsistent. For example, in the United States, a longitudinal study conducted in Chicago, Illinois suggested that neighborhood disorder does not directly promote crime (27); however a study conducted in Philadelphia, Pennsylvania found that women facing greater disadvantages were notably more worried about crime and safety in their neighborhoods. They also reported higher levels of perceived physical and social disorder compared to women with more advantages (28).

Neighborhood disorder and crime has been explored through its association with collective efficacy (CE) (27, 29–31). CE is a form of social capital that pertains to residents of a neighborhood with shared beliefs and their willingness to improve the neighbourhood through engaging in informal social control (27, 31). Higher levels of CE have been associated with lower levels of crime in the United States (27, 32). Findings from a study conducted in Chicago, Illinois suggest that reducing disorder may indirectly reduce crime by stabilizing neighborhoods through CE (27). In neighborhoods where there is strong CE, residents perceived crime and disorder as low (27). Furthermore, regardless of disorder and socioeconomic status, neighbourhoods with strong CE resulted in reduced violence (27). In addition to reducing crime and disorder, high levels of CE and social cohesion in neighbourhoods have been negatively associated with depression (33–35). A multilevel study conducted in New York, USA found that older adults (65 years and older) would have 6.2% lower prevalence of depression if they had lived in neighborhoods with high CE (34). Additionally, neighbourhoods with lower social cohesion showed higher rates of depression (35).

**Figure 1:**
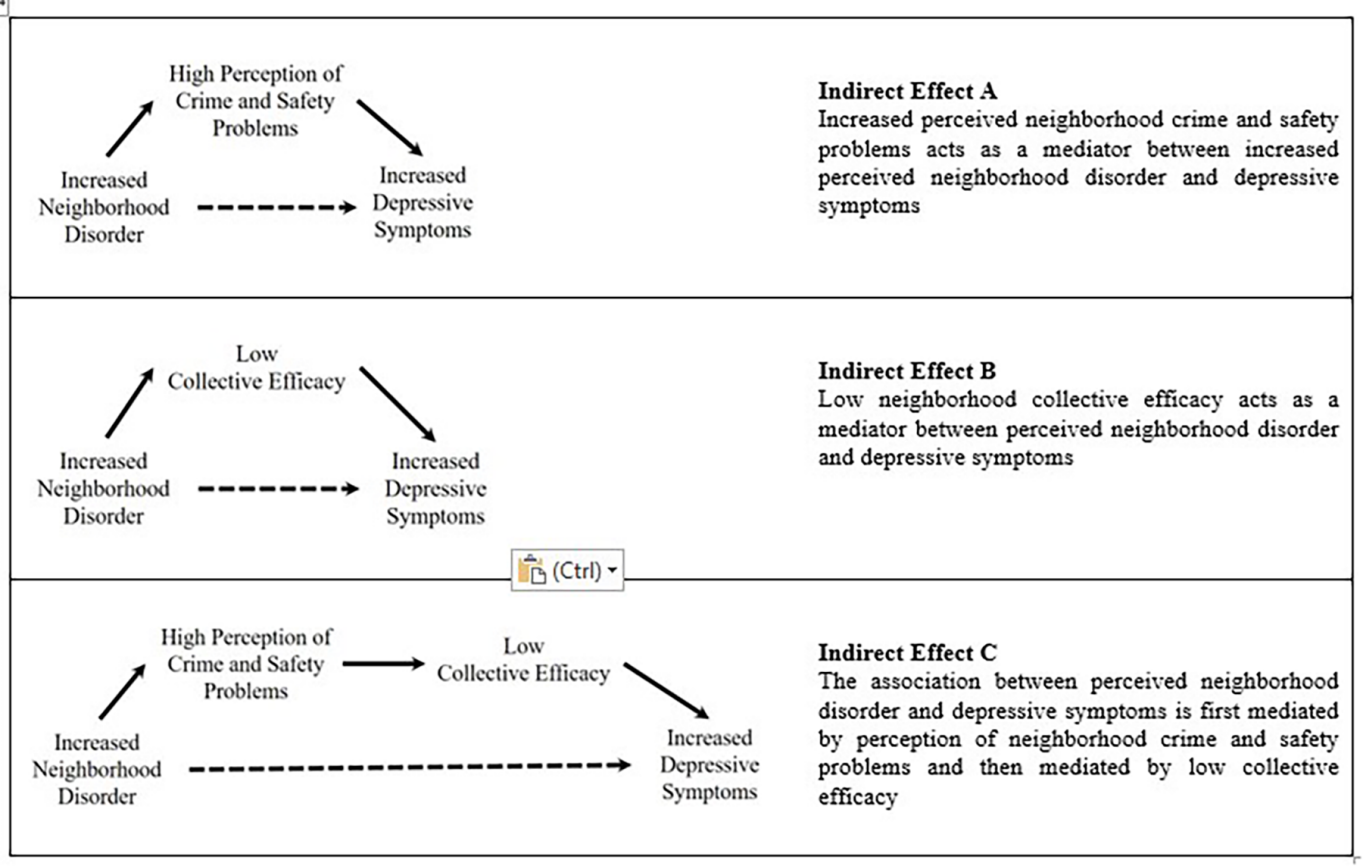
Hypothesized mediation pathways between neighborhood disorder and depressive symptoms.

Factors of social and physical disorder have negative mental health consequences for Jamaican residents (15). Nevertheless, gaps remain in our understanding of the role that neighborhood conditions may play in depressive symptomatology, particularly in middle-income small island developing states, like Jamaica. The aims of this paper were firstly, to examine the association of the perception of neighborhood disorder on depressive symptoms in a nationally representative sample of Jamaican adults and secondly, to evaluate the separate and serial mediating roles of perceived neighborhood crime and safety problems, as well as, CE in this association. We explored three mediation models: (i) whether perceived neighborhood crime and safety problems might work as a mediator between perceived neighborhood disorder and depressive symptoms [Indirect Effect A] (ii) whether CE mediates the association between perceived neighborhood disorder and depressive symptoms [Indirect Effect B] and (iii) serial mediation—whether the association between perceived neighborhood disorder and depressive symptoms is mediated by perception of neighborhood crime and safety problems which, in turn, is mediated by reported CE [Indirect Effect C]. Our conceptual models are presented below in Figure 1.

## 2 Materials and methods

### 2.1 Study design and sampling

Data were obtained from the Jamaica Health and Lifestyle Survey (JHLS III), a community based, cross-sectional face-to-face interviewer-administered survey of free-living Jamaican residents (*n* = 2,889) aged 15 years and older who were 95.2% Black (4). It was conducted between September 2016 and March 2017 and had a response rate of 89.0%. The survey determined the prevalence of chronic non-communicable diseases and risk factors inclusive of mental health disorders such as depression and perception of neighborhood characteristics. Trained interviewers recruited participants based on a stratified, random, two-stage cluster design. Firstly, enumeration districts (EDs), the primary sampling units, were selected randomly from parishes to reflect the urban-rural distribution of each of Jamaica's 14 parishes. Secondly, households were selected without replacement by systematic, random sampling from a list of dwellings within each ED, to obtain a nationally representative sample. The neighbourhood consisted of two or more contiguous EDs which had a minimum of 80 dwellings. Additional details on the survey methodology are provided in the full technical report (4).

### 2.2 Measures

#### 2.2.1 Outcome: depressive symptoms

The primary outcome, depressive symptoms, was defined as the presence of 5 or more symptoms of depression and/or expressions of suicidal ideation based on the Diagnostic and Statistical Manual of Mental Disorders versions 4/5 (DSM 5) (36). Participants were asked whether during the past month they had been bothered a lot by the following: (a) little interest or pleasure in doing things, (b) feeling down, depressed or hopeless, (c) feeling sad or lonely, (d) feeling guilty or worthless, (e) change in appetite, (f) change in sleeping patterns and (g) suicidal ideations—ever seriously considered suicide, how recently was suicide was considered, ever made a plan to commit suicide and ever attempted suicide (4).

#### 2.2.2 Exposure: perceived neighborhood disorder

The primary exposure, perceived neighborhood disorder, was measured using both the perception of physical and social disorder in the respondent's community. Perceptions of physical disorder were captured based on five questions measuring whether litter/trash, abandoned cars, graffiti on buildings, vacant buildings and houses and yards not being kept up were “a problem” in the neighborhood (28). Perceptions of social disorder were assessed using four questions regarding a perception of a “problem” in the neighbourhood concerning public drunkenness, young adults hanging around, unemployed adults and gang activity (28). Questions were asked on a scale from 1 (“rarely”) to 5 (“frequently”) and were dichotomized as high based on “most times” or “frequently” vs. low or “sometimes”, “a little”, or “rarely”. The variable neighbourhood disorder was created by summing the dichotomized high and low categories of the individual dichotomized physical and social disorder scores as well as overall high and low disorder based on whether respondents were high on either or both physical or social disorder items.

#### 2.2.3 Mediator: perception of neighborhood crime and safety problems

Perception of neighbourhood crime and safety problems was measured by seven questions regarding how worried the respondent was about crime and drug activity in their neighborhood. These included concerns about whether drug dealers/users were hanging around, property being stolen, walking alone in the daytime, letting children go outside during the day or night and being robbed or murdered (28).

#### 2.2.4 Mediator: collective efficacy

Collective efficacy, a component of social capital, was measured and included the mutual trust and shared expectations among neighbors or communities. It is measured using two subscales (1) social cohesion, the relationships between neighbors and (2) informal social control, community pressure for norms and laws. Each subscale included five items. For the social cohesion subscale, participants’ perceptions were obtained on whether their residential neighborhood was close-knit, had residents willing to help their neighbors and could be trusted, as well as, whether residents generally didn't get along or share the same values. The items on the construct of informal social control dealt with participants’ assessment of how likely neighbors were to intervene if children were skipping school, spray-painting graffiti, showing disrespect to adults or break up a fight; how likely residents would organize to keep the closest post office open if budget cuts threatened its closure was also assessed. The two subscales used, represented a minor adaptation of the original and validated scale by Sampson et al. (31).

#### 2.2.5 Potential confounders: sociodemographic characteristics

Sociodemographic characteristics were selected based on *a priori* theory. Included were sex, age, education (primary school or lower vs. secondary school or higher), employment as a proxy for socioeconomic status (SES) in three categories [unemployed, other (student or retired) and employed], and urban/rural residence.

### 2.3 Statistical analysis

The analyzed sample size was 2,719. Analyses included descriptive, bivariate and multivariate regression, including mediation modeling utilizing the Process Macro (37). Descriptive data analysis estimated total survey-weighted means, proportions and 95% confidence intervals (CIs) as well as by depressive symptom status. Logistic regression was utilized to examine the association between perceived neighborhood disorder and high depressive symptomatology, as well as, with neighborhood perceived crime and safety problems and CE, whilst adjusting for key covariates. Mediation analyses, including serial mediation, with bootstrapping was run to examine the mediation by both perceived neighborhood crime and safety problems and CE in the neighbourhood disorder and depressive symptomology relation. If the 95% CI of the indirect effects did not contain 0, it indicated that the mediating effect was significant. Complete case analysis was the method used to deal with missing data.

## 3 Results

Table 1 gives prevalence of persons with depression symptoms by sample characteristics among Jamaicans 15 years and older. The overall prevalence of depressive symptoms in the Jamaican population was 14.29%, with a significantly higher prevalence of 18.41% among women (*p* < 0.001) compared to 10.01% among men. Employed persons had a statistically significantly (*p* < 0.01) lower prevalence of depression (11.5%) compared to those who were unemployed (18.32%) or among those classified in the “Other” category (16.24%). There was a significantly higher proportion (*p* < 0.05) of persons with depressive symptoms that perceived neighborhood disorder as high (16.42%) compared with those who perceived the neighborhood disorder as low (8.76%). Table 1 also indicates a significant difference in the proportion (*p* < 0.001) of persons suffering from depression that perceived high crime and safety problems in their neighborhoods (19.71%), compared with those who perceived the problems as low (11.04%). An opposite statistically significant trend was seen for the association of CE with depression (*p* < 0.05) whereby high levels of CE were associated with lower levels of depression in 12.16% of participants compared with 18.12% for those with low levels of CE in their neighborhoods.

**Table 1:** Weighted prevalence of depressive symptoms by sample characteristics (95% CI) of persons in the Jamaica health and lifestyle survey 2017 (JHLS III).

Characteristic	Depressive symptoms (*N* = 2,719) % (95% CI)
Neigborhood disorder^a^	
Low	8.76 (7.19, 10.34)
High	16.42 (14.32, 18.53)*
Sex	
Male	10.01 (7.77, 12.79)
Female	18.41 (16.02, 21.06)***
Age	
<18	10.53 (5.08, 20.56)
18–59	14.61 (12.54, 16.96)
≧60	13.68 (10.55, 17.57)
Education	
≤Primary school	14.16 (11.49, 17.32)
≥Secondary school	14.38 (12.07, 17.05)
Other	6.78 (2.63, 16.37)
Employment	
Unemployed	18.32 (15.28, 21.80)
Other^b^	16.24 (9.92, 25.47)
Employed	11.50 (9.42, 13.97)**
Urbanicity	
Rural	12.37 (10.46, 14.57)
Urban	15.76 (12,79, 19.27)
Perceived crime and safety problems	
Low	11.04 (9.09, 13.36)
High	19.71 (16.28, 23.66)***
Collective efficacy	
Low	18.12 (14.02, 23.11)
High	12.16 (9.95, 14.78)*
**TOTAL**	**14.29** (**13.21, 15.37)**

Statistically significant estimates are in bold.

CI, confidence interval.

* *p* < 0.05, **p < 0.01, ***p < 0.001.

^a^ Includes both neighborhood physical and social disorder.

^b^ Includes students and retirees.

The multiple regression analysis results are presented in Table 2. The odds of depressive symptomatology was approximately 55% as high among respondents living in neighborhoods perceived as having high levels of disorder compared to those living in neighborhoods perceived to have low levels of disorder. Model 1 [adjusted OR (aOR) = 1.55, 95% CI = 1.14, 2.10] includes adjustment for sex, age, education, employment and urbanicity. When compared to employed persons, individuals who stated that they were not unemployed, but classified into the “other” employment category (which include for e.g., students and retirees) have a significantly higher likelihood of depressive symptoms (aOR = 2.03, 95% CI = 1.04, 3.94), with adjustments for the covariates perceived crime and safety problems, sex, age, education and urbanicity. The relationship between neighborhoods perceived to have high disorder and depressive symptomatology remained statistically significant after additionally considering perceived neighborhood crime and safety problems plus CE (Model 3, aOR = 1.58, 95% CI = 1.02, 2.44). Perceived neighborhood crime and safety problems was also significantly associated with depressive symptomatology, with individuals living in neighborhoods perceived as having high crime and safety problems being 75% as likely to report depressive symptomatology compared to those living in neighborhoods with low crime and safety problems (Model 2, aOR = 1.75, 95% CI = 1.23, 2.51). We also observed partial mediation by perceived neighborhood crime and safety problems and CE in the perceived neighborhood disorder-depressive symptomatology relation, with 12% and 7% of the association between perceived neighborhood disorder and depressive symptoms explained through the paths of perceived neighborhood crime and safety problems and low CE, respectively. We also observed a significant serial mediation pathway between perceived neighborhood disorder→perceived neighborhood crime and safety problems→CE→depressive symptoms, with an indirect effect (*β* = 0.03, bootstrapped SE = 0.02, 95% CI = 0.003, 0.07).

**Table 2:** Adjusted odds ratio (OR) and 95% confidence intervals (95% CI) for associations between perceived neighborhood disorder and depressive symptoms (*N* = 2,719).

Characteristic	Model 1		Model 2		Model 3	
	aOR	(95% CI)	aOR	(95% CI)	aOR	(95% CI)
Neighborhood disorder^a^						
Low	Reference		Reference		Reference	
High	**1**.**55**	**(1.14, 2.10)** *	1.29	(0.91, 1.82)	**1**.**58**	**(1.02, 2.44)** *
Sex						
Male	Reference		Reference		Reference	
Female	**1**.**54**	**(1.09, 2.19)** *	**1**.**58**	**(1.07, 2.26)** *	1.38	(0.88, 2.17)
Age						
<18	2.61	(0.91, 7.46)	2.43	(0.83, 7.14)	1.36	(0.34, 5.39)
18–59	2.42	(0.81, 7.24)	2.13	(0.69, 6.61)	1.86	(0.44, 7.94)
≧60	Reference		Reference		Reference	
Education						
≤Primary school	2.36	(0.83, 6.76)	3.21	(0.99, 10.36)	0.84	(0.25, 2.80)
≥Secondary school	2.49	(0.91, 6.84)	3.07	(0.99, 9.44)	0.91	(0.29, 2.89)
Other	Reference		Reference		Reference	
Employment						
Unemployed	1.39	(0.99, 1.94)	1.41	(0.98, 2.02)	1.19	(0.76, 1.88)
Other^b^	**2**.**03**	(1.04, 3.94)**	**2**.**55**	(1.24, 5.22)**	1.57	(0.57, 4.34)
Employed	Reference		Reference		Reference	
Urbanicity						
Rural	Reference		Reference		Reference	
Urban	1.25	(0.91, 1.72)	1.16	(0.83, 1.62)	1.29	(0.84, 1.96)
Perceived crime and safety problems						
Low			Reference			
High			**1**.**75**	(1.23, 2.51)**		
Collective efficacy						
Low					Reference	
High					0.71	(0.44, 1.14)
**Mediated effect**			**β**	**(95% CI)**	**β**	**(95% CI)**
**High perceived crime and safety Problems**			**0**.**12**	(0.06, 0.17)**		
**Low collective efficacy**					**0**.**07**	(0.03, 0.14)**
**Serial mediation (disorder→high Perceived crime and safety problems→low CE**					**0**.**03**	**(0.003, 0.07)** *

Each model is adjusted for all the other variables listed.

Statistically significant estimates are in bold.

CE, collective efficacy, CI, confidence interval, OR, odds ratio.

* *p* < 0.01, **p < 0.05.

^a^ Includes both neighborhood physical and social disorder.

^b^ Includes students and retirees.

## 4 Discussion

This is the first study to examine the relationship between perceived neighborhood disorder and depression in Caribbean adults and whether perceived neighbourhood crime and safety problems, as well as, CE played a mediating role. We observed a significant association between neighborhood perceived disorder and self-reported depressive symptoms, as well as, potential indirect pathways through perceived neighborhood crime and safety problems and CE. In general, respondents who lived in areas perceived as high disorder were 1.6 times as likely to report high depressive symptomology and respondents who perceived neighborhood crime and safety problems in their neighbourhood as high were nearly twice (1.75 times) as likely to report high depressive symptomatology. In the perceived neighborhood disorder-depression/depressive symptomatology relationship, we observed partial mediation by the neighborhood characteristics of interest, with 12% and 7% of the association between perceived neighborhood disorder and depressive symptoms explained through the paths of high perceptions of neighborhood crime and safety problems and low CE, respectively. The hypothesized serial mediation pathway via high perception of neighborhood crime and safety problems and low CE was found to be significant. Our results indicate depressive symptoms were reported more frequently among women compared to men.

Our results align with prior strong evidence that perceived neighborhood disorder is positively associated with depressive symptomatology, even though there was variability in the definitions of neighbourhood and measurement of the construct of perceived neighborhood disorder, and differed from those in our study. For example, in the only study we found that examined a similar association among Jamaican adults, urban females in Jamaica were twice as likely to report depressive symptoms among those living in physically disordered neighborhoods (15). Similarly, in the study by Hastings and Snowden that included three nationally representative surveys of African American and Caribbean Blacks combined and residing in the USA (18), among individuals of low socioeconomic status, those who perceived neighborhood social disorder were 1.7 times as likely to report past-year depression.

Whilst the direct and positive association between perceived crime and safety problems and depression was consistent with our expectations, we did not observe a direct association between CE and depression as found in other studies (33–35, 37). A possible explanation is that these studies differed from our research methodology in one of, or a combination of factors, such as, the age groups of the participants, variables included in conceptual models, or the tools used to measure depression. Nonetheless, our findings extend the literature by confirming the mediating roles of both perceived neighborhood crime and safety problems, and CE, on the association between disorder and depressive symptomatology, with some of the former's impact acting via CE.

### 4.1 Strengths and limitations

The data from our study was retrieved from a national survey, which consisted of a nationally representative sample and hence, is generalizable across Jamaica and other societies with similar attributes. Although the data were collected almost 7 years ago, we have no theoretical reason to believe the results are time-dependent. Despite these important findings, this study is not without potential limitations. First, given the cross-sectional study design, results do not establish causality between perceived neighborhood disorder, perceived neighborhood crime and safety problems, or CE and depressive symptoms. A second limitation is that the study may be impacted by recall bias by participants when completing the survey. It has been recommended that using both objective and subjective neighborhood measures may eliminate some of the biases that may occur when examining associations between self-reported neighborhood exposures and self-reported outcomes (26). Thirdly, we did not ascertain the participants' perspectives and/or behaviours, as to why and how these neighbourhood factors may be driving the associations with depression. Future studies could include a qualitative component to gain additional insights, including narratives on the participants' perspectives and lived experiences.

### 4.2 Conclusions

Depression is a leading cause of disability and also a risk factor for many illnesses and diseases. There are many individual level factors that lead to depression however contextual factors are essential in understanding depression. Our findings, utilizing mediation studies, suggests that the pathway between neighborhood disorder and depressive symptoms may be reduced by intervening on reducing neighborhood crime and safety problems and/or improving CE in Jamaican neighborhoods.

## Data Availability

The datasets presented in this article are not readily available because data requests can be made to the Ministry of Health and Wellness Jamaica and will be reviewed on a case-by-case basis. Requests to access the datasets should be directed to Dr. Karen Webster-Kerr, karen.websterkerr@moh.gov.jm.
